# Challenges and dilemmas of exercise therapy in the management of osteoporosis: a narrative review

**DOI:** 10.3389/fendo.2026.1931476

**Published:** 2026-09-29

**Authors:** Jiancheng Yang, Lixin Zhang, Yuhong Zeng

**Affiliations:** 1 Department of Osteoporosis, Honghui Hospital, Xi’an Jiaotong University, Xi’an, China; 2 Department of Clinical Pharmacy, Honghui Hospital, Xi’an Jiaotong University, Xi’an, China

**Keywords:** bone mineral density, exercise therapy, fall prevention, osteoporosis, resistance training, vertebral fracture

## Abstract

Osteoporosis is a chronic, progressive bone condition that impacts over 200 million people globally, with fragility fractures significantly burdening healthcare systems and diminishing patient quality of life. Exercise is universally recommended as a foundational non-pharmacological intervention for osteoporosis management; however, a persistent translational gap separates the efficacy demonstrated in controlled trials from the outcomes observed in real-world clinical practice. This narrative review identifies and critically examines the multifaceted challenges confronting the evidence-based application of exercise therapy in contemporary osteoporosis care. Seven principal interrelated dilemmas are elucidated: the surrogate endpoint dependency on bone mineral density (BMD) rather than fracture incidence, profound heterogeneity of osteogenic effects across exercise modalities, the tension between effective osteogenic thresholds (≥70% 1RM) and safety concerns in high-risk subgroups, the absence of validated patient stratification tools, poor real-world adherence amid scarce supervised infrastructure and fragmented care pathways, pervasive nutritional inadequacies that undermine the skeletal response to mechanical loading, and methodologically heterogeneous evidence for traditional mind-body exercises that limits their equivalence to progressive resistance training. The reversibility of bone gains upon detraining further underscores that exercise must be conceptualized as a lifelong behavior rather than a time-limited intervention. Future priorities include fracture-powered pragmatic trials, validated osteoporosis-specific pre-exercise risk stratification instruments, implementation science addressing behavioral and systemic barriers, and integrated multidisciplinary care models that unite exercise professionals with bone specialists and dietitians. Until such evidence matures, clinicians must navigate a landscape of conditional recommendations, individualized risk-benefit assessments, and honest communication of uncertainty to patients.

## 1 Introduction

Osteoporosis is a widespread bone condition marked by reduced bone density and the breakdown of bone structure, leading to an increased risk of fragility fractures (1, 2). An estimated 200 million people around the world suffer from osteoporosis, which results in approximately 9 million fragility fractures every year (3, 4). With the progressive aging of populations across both developed and developing nations, the occurrence of osteoporosis and the associated risk of fractures are expected to increase significantly in the next few decades. The consequences of these fractures extend far beyond the acute event. Hip fractures, in particular, are linked to mortality rates of 16.6%, 25.3%, and 33.9% within 1, 2, and 3 years, respectively (5). Vertebral fractures, while often clinically silent, contribute to progressive thoracic kyphosis, chronic pain, reduced pulmonary function, and increased risk of subsequent fractures (6). The cumulative burden of osteoporotic fractures on healthcare systems, caregivers, and society at large underscores the urgent need for effective, accessible, and sustainable prevention and management strategies.

While pharmacological interventions, including bisphosphonates, denosumab, teriparatide, and the more recently approved romosozumab, form the mainstay of fracture risk reduction, non-pharmacological strategies are increasingly recognized as indispensable adjuncts to comprehensive osteoporosis management (7–10). Among these, exercise has emerged as a key intervention with unique and potentially synergistic advantages: it is low-cost, non-invasive, generally well tolerated when appropriately prescribed and supervised, and capable of simultaneously addressing multiple fracture risk determinants, including bone density, muscle strength, balance, and functional capacity. Indeed, the International Osteoporosis Foundation (IOF) and the World Health Organization (WHO) have both identified physical activity as a first-line preventive strategy for osteoporosis, and national guidelines across Europe, North America, and Asia universally recommend exercise as an integral component of bone health promotion (11, 12).

The rationale for exercise in osteoporosis is firmly grounded in biomechanical and cellular biology. Bone tissue is exquisitely sensitive to mechanical strain; osteocytes, embedded within the mineralized matrix, function as mechanosensors that transduce deformation signals into anabolic remodeling directives via the Wnt/β-catenin pathway and the osteoprotegerin (OPG)/receptor activator of nuclear factor-kappa B ligand (RANKL) signaling axis (13, 14). When mechanical loading generates strain magnitudes and rates that exceed habitual thresholds, these pathways favor bone formation over resorption, thereby preserving or augmenting bone mineral density (BMD) and improving bone geometry and microarchitecture (15, 16). Furthermore, physical activity that generates supra-threshold ground reaction forces and muscular tension has the theoretical capacity to improve muscle strength, neuromuscular coordination, proprioception, and postural control, thereby reducing falls—a major proximal cause of fragility fractures in older adults (17, 18). The mechanostat theory, originally proposed by Harold Frost, posits that bone adapts its mass and structure to the habitual mechanical demands placed upon it; consequently, when mechanical loading is reduced—as in sedentary behavior, prolonged bed rest, or microgravity environments—bone resorption outpaces formation, leading to rapid bone loss (19, 20). Conversely, targeted exercise that imposes controlled, progressive mechanical overload may induce a net positive balance in bone remodeling, particularly in individuals whose habitual activity levels are below the osteogenic threshold.

Despite this robust biological rationale, the clinical translation of exercise science into routine osteoporosis care is far from straightforward. The contemporary literature is voluminous yet fragmented: hundreds of randomized controlled trials, dozens of systematic reviews and meta-analyses, and numerous international consensus statements have been published over the past two decades. Yet healthcare professionals and patients remain confronted with profound and persistent uncertainty. Clinicians routinely ask: What type of exercise is most effective for a specific patient? What intensity is safe for an individual with prior vertebral fractures? How can bone gains be sustained after the supervised intervention ends? Why do real-world adherence rates fall so far short of the benchmarks reported in clinical trials? How should traditional mind-body exercises such as Tai Chi and Baduanjin be positioned within an evidence-based hierarchy that is increasingly dominated by progressive resistance training and high-impact protocols? These questions do not reflect the failure of exercise as a therapeutic modality; rather, they expose a series of deep-seated challenges and dilemmas that pervade the evidence base, the clinical guidelines, and the healthcare delivery systems that mediate between them.

This narrative review was undertaken with the aim of exploring these challenges and dilemmas, clarifying the boundaries of current understanding, identifying gaps in methodology and translation, and articulating priorities for future research and clinical practice. Drawing on published evidence from systematic reviews, meta-analyses, randomized controlled trials (RCTs), international consensus statements, and national clinical guidelines, we aim to deliver a detailed, evidence-based, and clinically relevant framework for understanding the current state and limitations of exercise therapy in managing osteoporosis.

## 2 Efficacy-evidence gaps: surrogate endpoints and heterogeneous outcomes

### 2.1 The BMD–fracture translation problem

The most fundamental and pervasive dilemma in the osteoporosis exercise literature is the near-exclusive reliance on BMD as the primary efficacy endpoint for exercise interventions. BMD, as measured by dual-energy X-ray absorptiometry (DXA), is a validated surrogate for fracture risk at the population level: each standard deviation decrease in BMD is associated with approximately a two-fold increase in fracture risk, and BMD-based diagnostic criteria (T-score ≤ −2.5) are the foundation of the WHO definition of osteoporosis (21). However, BMD explains only a fraction, estimated at 60%–70% of bone strength variance (22). The remaining proportion is attributable to bone quality, encompassing microarchitecture, cortical and trabecular geometry, matrix composition, mineralization, and the accumulation of microdamage (23, 24). These dimensions of bone quality are not captured by areal BMD measurements, yet they are critical determinants of fracture resistance. Consequently, interventions that improve BMD may not necessarily reduce fracture risk proportionally, and conversely, interventions that enhance bone quality without significantly altering BMD may confer meaningful anti-fracture benefits that are not detected by conventional densitometry.

In the exercise literature, meta-analyses and systematic reviews have consistently documented modest but statistically significant BMD improvements with progressive resistance training (PRT) and impact exercise. A meta-analysis of 80 randomized controlled trials (RCTs) in postmenopausal women reported standardized mean differences (SMDs) of approximately 0.29 (95% confidence interval [CI]: 0.16–0.42) at the lumbar spine and 0.27 (95% CI: 0.16–0.39) at the femoral neck (25). When PRT is combined with high-impact exercise, the SMDs increase to 0.41 (95% CI: 0.18–0.65) at the femoral neck and 0.43 (95% CI: 0.16–0.70) at the lumbar spine (26). A network meta-analysis of 49 RCTs further reinforced this hierarchy, demonstrating that combined resistance and impact training produced the largest effect sizes for both lumbar spine and femoral neck BMD, followed by resistance training alone and high-impact exercise alone (27). These effect sizes, while statistically significant, translate into relatively small absolute changes in BMD—typically less than 1%–2% at any single site. Whether such increments independently and clinically meaningfully reduce fracture incidence remains uncertain. Although BMD is a strong predictor of fracture risk in observational cohorts, it has not been formally validated as a surrogate endpoint for the anti-fracture efficacy of exercise in the context of clinical trials.

It is important to distinguish between two levels of evidence. On one hand, pooled meta-analysis evidence from controlled trials suggests that exercise may reduce the incidence of low-trauma and major osteoporotic fractures in middle-aged and older adults (28). On the other hand, no single adequately powered RCT has yet demonstrated that exercise alone reduces fragility fracture incidence specifically in older adults with established osteoporosis who are eligible for pharmacological therapy. The logistical and financial barriers to such fracture-powered exercise trials are formidable: fracture rates in community-dwelling older adults are low, requiring tens of thousands of participants and years of follow-up to achieve adequate statistical power (29). Moreover, the ethical considerations of withholding established pharmacological therapy from high-risk individuals complicate the design of exercise-only trials. An RCT investigating high-dose cholecalciferol supplementation combined with long-term physical therapy in patients recovering from hip fractures (30) illustrates the complexity of isolating exercise effects. Although it included a physical therapy component, it was not designed as an exercise-only fracture-prevention trial and was underpowered for its primary endpoint. Consequently, although meta-analytic pooling provides encouraging evidence for fracture reduction, the anti-fracture efficacy of exercise as a standalone intervention in the osteoporosis population remains inferred rather than directly proven through adequately powered RCTs. This evidentiary limitation complicates the communication of clinical benefits to patients, many of whom are understandably more concerned with avoiding fractures than with improving their T-score.

The implications of this BMD-fracture translation problem are broad. For clinicians, it means that exercise recommendations must be framed as conditional: “exercise is likely to improve bone health and may reduce fracture risk, but we cannot guarantee this with the same certainty as pharmacological treatments.” For researchers, it underscores the urgent need for fracture-powered trials, possibly leveraging pragmatic trial designs that incorporate exercise as an adjunct to rather than a replacement for standard pharmacological care. For policymakers, it raises questions about the evidentiary threshold required to justify reimbursement and resource allocation for exercise-based osteoporosis programs. Until fracture endpoints are established, the field must operate with acknowledgment of uncertainty that is often absent from clinical guideline language.

### 2.2 Heterogeneity of outcomes across exercise modalities

Not all exercise modalities exert equivalent osteogenic effects, and the heterogeneity of outcomes across different types of physical activity represents a second major challenge for evidence-based exercise prescription. This heterogeneity is both a source of scientific confusion and a clinical dilemma, as clinicians must navigate a landscape of conflicting or incomplete recommendations without clear guidance on which modality to prioritize for a given patient. **Figure 1** presents the comparative osteogenic efficacy of eight exercise modalities versus no exercise in postmenopausal women with osteoporosis or osteopenia, as reported in a recent network meta-analysis of 49 randomized controlled trials (27). The complete data, including effect measures, 95% confidence intervals, and the number of participants contributing to each node, are available in **Supplementary Table S1**. At the lumbar spine, combined aerobic and resistance training [AE + RT; mean difference (MD) = 32.35, 95% CI: 8.08–56.62], aerobic exercise (AE; MD = 22.33, 95% CI: 6.67–37.99), and resistance training (RT; MD = 16.38, 95% CI: 5.03–27.73) significantly enhanced BMD when compared with the control group. Significant effects on BMD improvement at the femur neck were found when comparing exercise types of AE + RT (MD = 140.00, 95% CI: 40.89–239.11), whole-body vibration (WBV; MD = 26.07, 95% CI: 2.97–49.16), and RT (MD = 16.98, 95% CI: 8.98–24.99) to the control group. Other activities such as Tai Chi (TC), impact exercise (IE), and walking did not demonstrate statistically significant BMD improvements versus control at either skeletal site in this analysis. In a pivotal review, Cauley and Giangregorio concluded that walking or TC alone is insufficient for preserving hip BMD and has a modest effect on spinal BMD (29).

**Figure 1:**
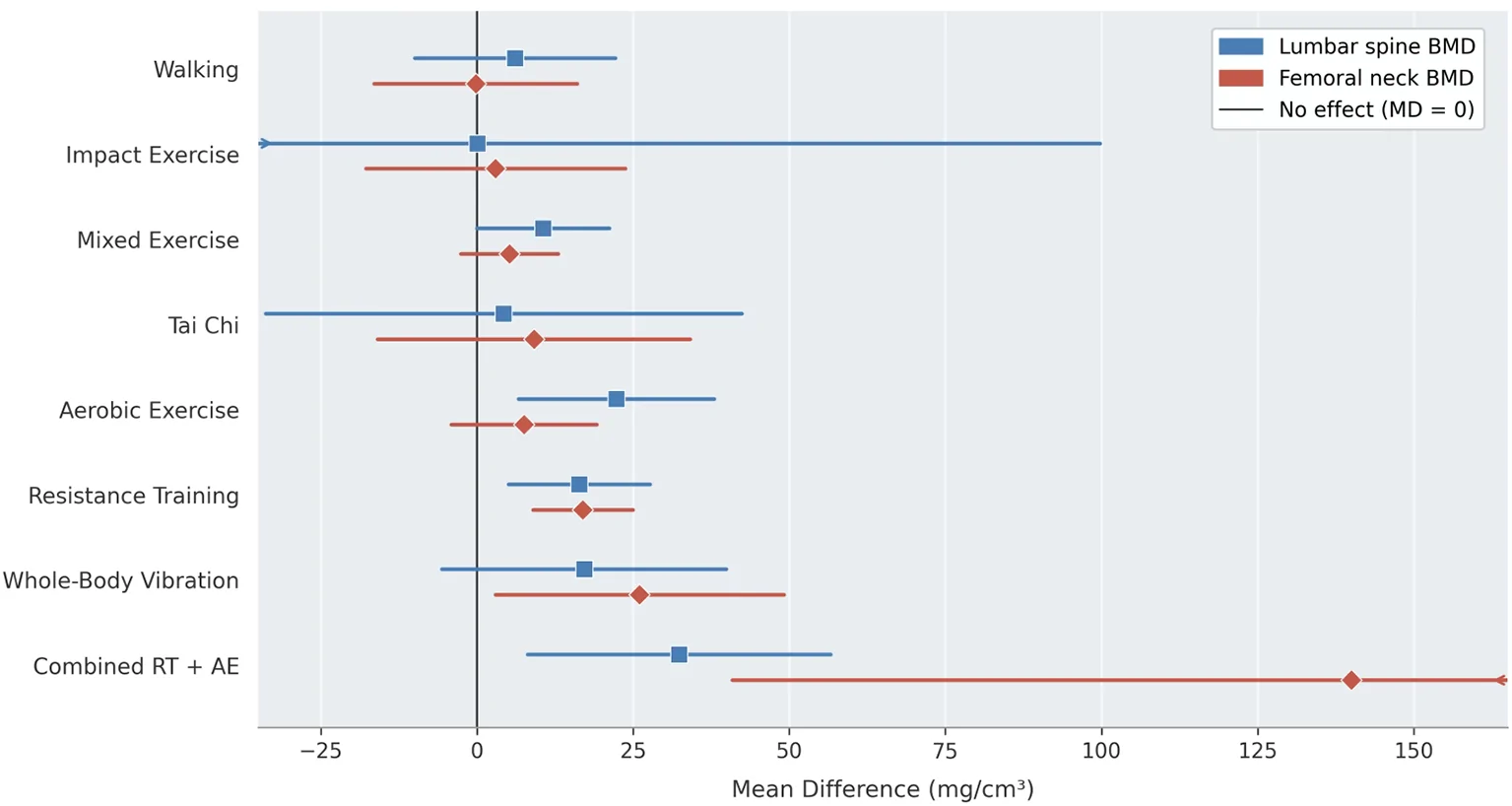
Comparative osteogenic efficacy of eight exercise modalities versus a no-exercise control in postmenopausal women with osteoporosis or osteopenia. Data are derived entirely from a single network meta-analysis of 49 randomized controlled trials (n = 3,360) by Xiaoya et al. (27). Effect estimates are presented as mean differences (MD) with 95% confidence intervals. BMD, bone mineral density; AE + RT, combined aerobic and resistance training.

It is important to emphasize that the non-significant estimate for impact exercise in **Figure 1** reflects the findings of this specific network meta-analysis and should not be interpreted as evidence that impact-based loading is osteogenically ineffective overall. A systematic review and meta-analysis specifically examining impact exercise in adults aged 50 years or older with low bone mass or fragility fractures found that impact exercise alone significantly improved lumbar spine BMD and total hip BMD, although the certainty of evidence was rated low (31). At the femoral neck, high-impact exercise alone or combined with resistance training also produced a significant pooled effect. These findings illustrate that conclusions regarding the osteogenic efficacy of impact exercise are sensitive to network configuration, evidence node density, and the specific analytical framework employed, and that a non-significant estimate in one network meta-analysis should not be overextended to imply general ineffectiveness.

Whole-body vibration (WBV) exemplifies the uncertainty surrounding emerging modalities. The theoretical rationale is appealing: high-frequency, low-amplitude mechanical oscillations are transmitted through the skeletal system, generating micro-strains that may stimulate osteocyte activity. However, the empirical evidence is inconsistent. Some meta-analyses have reported small positive effects on lumbar spine BMD under specific parameter conditions (approximately 30 Hz, low amplitude, high cumulative dose) (32), while others have found no significant benefit on BMD at either the femoral neck or lumbar spine (33). The 2024 UK clinical guidelines recommend WBV solely as a fall prevention measure and do not endorse its use for increasing BMD (7), reflecting insufficient evidence maturity. The variability in WBV parameters across studies, such as frequency, amplitude, duration, platform type, and participant posture, makes systematic synthesis and clinical translation exceptionally difficult.

The heterogeneity of outcomes is not merely a matter of different effect sizes; it reflects fundamentally different mechanisms of action. Resistance training primarily generates osteogenic strain through muscle tension and joint reaction forces, which are particularly effective at load-bearing sites such as the femoral neck and lumbar spine. Impact exercise, such as jumping and hopping, generates high-magnitude, high-rate ground reaction forces that are especially potent at stimulating cortical bone adaptation. Balance and proprioceptive training, by contrast, exert minimal direct osteogenic effects but may reduce fracture risk indirectly by preventing falls. Tai Chi and other traditional exercises may improve BMD through a combination of low-intensity loading, weight shifting, and neuromuscular training, but the magnitude of effect is consistently smaller than that of progressive resistance training. Swimming, although an excellent form of aerobic exercise, is osteogenically neutral or even slightly negative at the hip in some populations, as the buoyancy of water eliminates the gravitational loading necessary for bone adaptation (34). This mechanistic diversity means that a “one-size-fits-all” exercise prescription is neither scientifically justified nor clinically optimal. Clinicians must consider the patient’s specific fracture risk profile, functional status, comorbidities, preferences, and access to resources when selecting an exercise modality, yet current guidelines provide only broad directional recommendations rather than precision-based algorithms.

Furthermore, the reporting of exercise outcomes is itself heterogeneous. Studies vary in their choice of BMD measurement sites (lumbar spine, femoral neck, total hip, trochanter, Ward’s triangle), imaging modalities (DXA, quantitative computed tomography [QCT], peripheral quantitative computed tomography [pQCT]), and outcome metrics (areal BMD, volumetric BMD, bone mineral content, bone area). This methodological heterogeneity limits the comparability of effect sizes across studies and complicates meta-analytic synthesis. For example, QCT-derived volumetric BMD may be more sensitive to cortical bone changes than DXA-derived areal BMD; however, few exercise studies employ QCT due to its higher cost and radiation dose (35). The field would benefit from standardized outcome reporting guidelines specific to exercise intervention trials, yet no such consensus currently exists.

## 3 The intensity paradox: effective thresholds versus safety boundaries

### 3.1 The intensity threshold for osteogenic adaptation

A central and persistent dilemma in exercise prescription for osteoporosis is the tension between the stimulus intensity required to produce meaningful bone adaptation and the tolerability and safety of that intensity for a diverse patient population. Mechanical loading theory, supported by extensive *in vivo* and ex vivo evidence, posits that bone adaptation requires strain magnitudes and rates that exceed habitual levels (36). This principle is consistent with the mechanostat model: bone tissue maintains a homeostatic set point for strain, and deviations from this set point in either direction trigger compensatory remodeling (19). When habitual loading is low, the set point is downregulated, and bone mass is lost. When supra-threshold loading is applied, the set point is upregulated, and bone formation is favored. However, the precise threshold for osteogenic adaptation in humans, particularly in older adults with compromised bone health, remains imprecisely defined.

Meta-analytic evidence provides important but incomplete guidance. Kistler-Fischbacher et al. (37) found that high-intensity (>80% 1RM) exercise produced the largest mean difference for lumbar spine BMD, followed by moderate-intensity (65%–80% 1RM) and low-intensity (<65% 1RM) exercise. However, at the femoral neck, low and moderate intensities demonstrated comparable significant effects, whereas high-intensity effects did not reach statistical significance—likely attributable to the paucity of high-intensity trials (n = 4) and insufficient spread in training intensities for meta-regression. Crucially, meta-regression revealed no significant association between resistance training intensity (expressed as %1RM) and BMD changes at either the lumbar spine or femoral neck (37). A subsequent network meta-analysis identified moderate-intensity resistance training as the optimal protocol for lumbar spine, femoral neck, and total hip BMD, with surface under the cumulative ranking curve (SUCRA) values exceeding those of both low- and high-intensity protocols (38). This apparent discrepancy is largely attributable to methodological differences: the high-intensity superiority reported by Kistler-Fischbacher et al. rests on a pairwise meta-analysis of only four interventions, rendering that estimate unstable, whereas the moderate-intensity optimality reported by Wang et al. derives from a network meta-analysis that permitted direct head-to-head comparison across protocols with more balanced evidence nodes. In addition, Wang et al. restricted inclusion to resistance-only training, whereas Kistler-Fischbacher et al. pooled all exercise modalities, meaning that their high-intensity group likely captured the synergistic effects of combined resistance and impact training rather than intensity per se. Overall, moderate-to-high intensity may represent the most evidence-supported prescription window (**Figure 2**). It is important to note that **Figure 2** merely presents a conceptual schematic synthesizing qualitative conclusions from meta-analyses and RCTs (15, 37, 38). This figure is intended as a heuristic framework to guide clinical reasoning rather than as a quantified dose-response curve. Moreover, even within the moderate-to-high intensity window, the osteogenic response is modulated by a constellation of interacting factors that are rarely isolated in clinical trials, including exercise selection (multi-joint versus single-joint, impact versus non-impact), skeletal site, loading rate and direction, training volume, progression schema, supervision quality, baseline physical activity, adherence, nutritional status (particularly calcium and protein adequacy), and concurrent pharmacotherapy. The relative contribution of each factor remains poorly quantified, and the intensity thresholds discussed above should be interpreted as approximate guides rather than rigid prescriptions.

**Figure 2:**
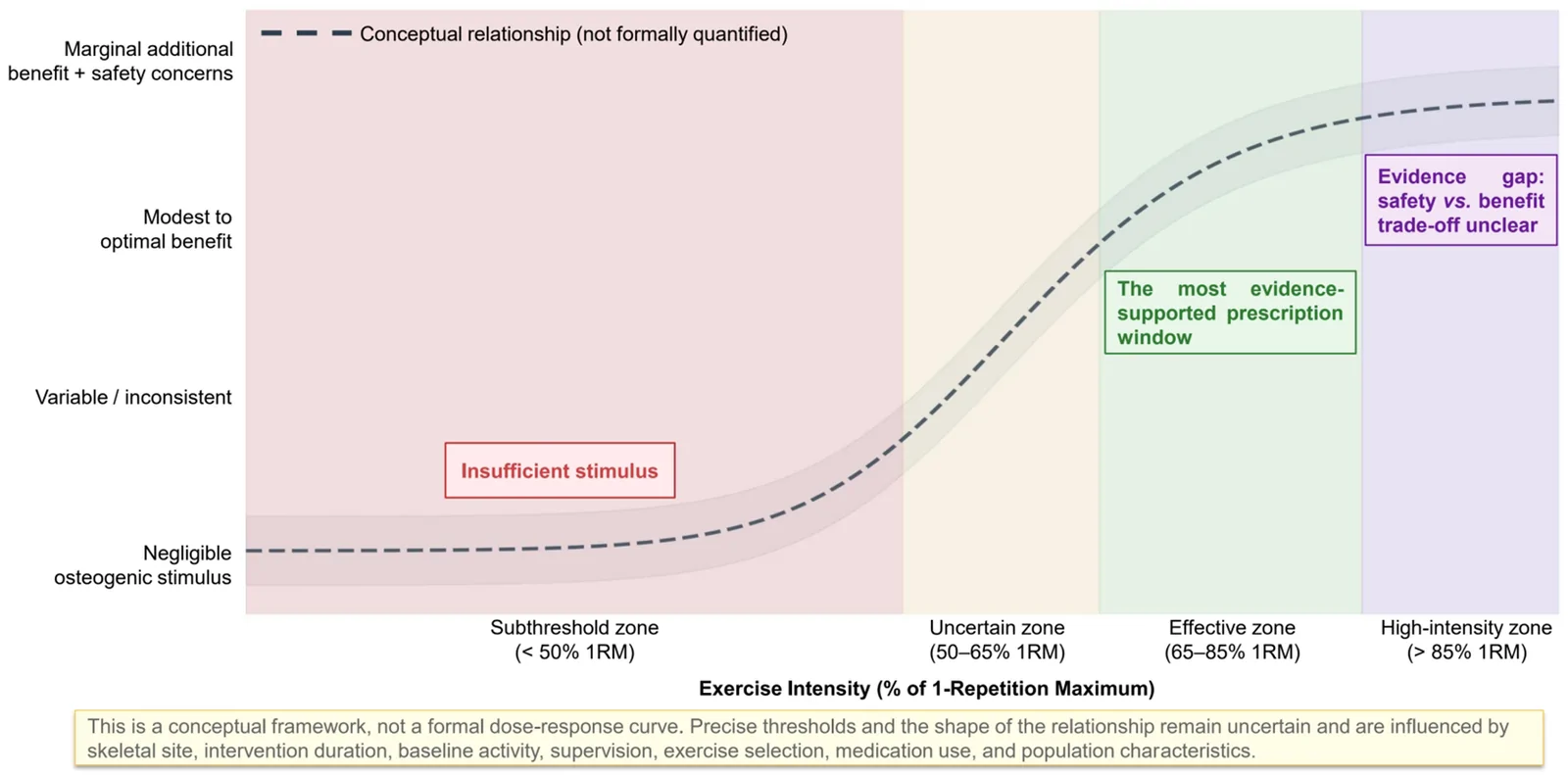
The intensity paradox in resistance training for osteoporosis. The solid curve depicts the conceptual estimated dose-response relationship between exercise intensity (% of one-repetition maximum, 1RM) and bone mineral density (BMD) effect size, synthesized from published meta-analyses and landmark RCTs regarding exercise intensity and BMD outcomes (15, 37, 38). The subthreshold zone (<50% 1RM) is associated with negligible osteogenic stimulus; the uncertain zone (50%–65% 1RM) shows variable responses; the effective zone (65%–85% 1RM) corresponds to the intensity range used in trials with significant BMD improvements. The high-intensity zone (>85% 1RM) may offer marginal additional BMD benefit but introduces safety concerns, particularly for individuals with prior vertebral fractures, that remain insufficiently quantified by empirical data.

Despite the analytical limitations observed in some comparative networks, impact exercise—particularly when combined with progressive resistance training—has demonstrated measurable osteogenic benefits across multiple skeletal sites. The landmark LIFTMOR trial, published in 2018, provided the strongest empirical evidence to date for the efficacy and safety of high-intensity resistance and impact training (HiRIT) in postmenopausal women with low bone mass (15). In this study, 8 months of twice-weekly, supervised HiRIT (>85% 1RM for resistance exercises, and approximately 50 multidirectional jumps per session) improved lumbar spine BMD by approximately 2.9 percentage points and maintained femoral neck BMD (+0.3%), whereas the control group experienced losses of 1.2% and 1.9%, respectively, at these sites. The LIFTMOR trial was methodologically rigorous, with blinded outcome assessment, intention-to-treat analysis, and a robust safety monitoring protocol. However, its generalizability is limited by the selection criteria: participants were “otherwise healthy” volunteers, excluded if they had uncontrolled cardiovascular disease, severe mobility limitation, or recent fractures. Whether such intensity is appropriate, acceptable, or even feasible for the broader osteoporosis population, which includes many individuals who are frail, multimorbid, physically inactive, and fearful of injury, remains unclear.

Recent evidence suggests that the translational challenges surrounding HiRIT extend beyond efficacy and safety to intervention reproducibility. A systematic review of 39 studies involving 3,349 peri- and postmenopausal women found moderate-to-good overall completeness of exercise reporting (mean Consensus on Exercise Reporting Template [CERT] score: 14.3 of 19), but recurrent omissions regarding loading progression, individualization, home-based components, and behavioral support strategies (39). Such deficiencies are not merely methodological niceties; they limit replication across clinical settings and hinder the translation of efficacious trial protocols into reproducible, implementable exercise prescriptions. When clinicians attempt to replicate supervised laboratory protocols in community or home settings, the absence of detailed reporting on progression algorithms, tailoring criteria, and adherence support leaves them without the necessary guidance to preserve intervention fidelity. Accordingly, future HiRIT trials should adopt standardized exercise-reporting frameworks, particularly the CERT, and provide sufficient detail regarding loading progression, supervision structure, individualization strategies, adherence support, and safety modifications to enable faithful replication.

### 3.2 Safety boundaries in high-risk subgroups

The safety limits of high-intensity and high-impact exercises are particularly controversial and unclear for those with previous vertebral fractures or several low-trauma fractures. Vertebral fractures are the most frequent type of osteoporotic fracture and are associated with a fivefold higher risk of subsequent vertebral fractures during the initial year in postmenopausal women (40). The structural integrity of the vertebral column is compromised, and the mechanical loading of the spine during exercise, particularly during flexion, rotation, and impact activities, raises legitimate concerns about fracture exacerbation or new fracture occurrence.

The “Too Fit to Fracture” consensus explicitly stated that general physical activity guidelines do not apply to individuals with prior vertebral fractures and that moderate intensity should be emphasized (41). The 2022 UK consensus statement advised limiting impact exercises to brisk walking levels for individuals with vertebral fractures and avoiding prolonged, repetitive, or extreme spinal bending (42). The American Association of Clinical Endocrinologists (AACE) and American College of Endocrinology (ACE) 2020 guidelines listed specific contraindicated movements: spinal flexion, trunk rotation under load, and heavy lifting (43). While these recommendations are reasonable, it is essential to acknowledge that they are primarily based on consensus rather than empirical data. The evidence base for exercise safety in individuals with prior vertebral fractures is sparse, consisting primarily of small, uncontrolled studies and expert opinion.

Nevertheless, emerging evidence from carefully selected, closely supervised exercise cohorts suggests that physical activity can be implemented without excess harm in certain subgroups of individuals with vertebral fractures. A meta-analysis of 19 studies reported that exercise intervention adverse event rates in individuals with vertebral fractures were only 6%, comparable to control conditions, and that no study reported an increase in vertebral fracture incidence attributable to the intervention (44). Similarly, a secondary analysis of the LIFTMOR trial cohort found that supervised HiRIT improved thoracic kyphosis without causing new vertebral fractures over the 8-month intervention period in postmenopausal women with low to very low bone mass (45). However, these reassuring findings must be interpreted with important caveats. The available trials have predominantly enrolled participants who were medically screened, progressively familiarized with loading, and supervised by qualified exercise professionals; sample sizes have been small; and rare serious adverse events inherently require very large populations to quantify reliably. Consequently, the highest-risk subgroups, those with severe osteoporosis (T-score ≤ −3.0), multiple vertebral fractures, substantial kyphotic deformity, frailty, acute pain, or multiple comorbidities, remain underrepresented. The absence of reported vertebral fractures in these selected cohorts therefore does not establish the safety of high-intensity exercise across all individuals with prior vertebral fractures, and generalization to the broad osteoporosis population should be avoided.

The dilemma, therefore, is not whether these patients should exercise, but how to operationalize intensity titration without validated pre-exercise risk stratification tools. Currently, no osteoporosis-specific preparticipation screening instrument has been widely validated to prospectively predict exercise-related adverse events and guide individualized intensity prescription. Clinicians must extrapolate from generic cardiovascular screening algorithms such as the ACSM 2015 screening protocol, which was designed for general populations and may be overly conservative for some individuals and insufficiently specific for others (46). The development of a validated, osteoporosis-specific pre-exercise assessment tool that integrates bone density, fracture history, vertebral morphology (e.g., Genant semiquantitative grading), functional capacity (Timed Up and Go [TUG], Short Physical Performance Battery [SPPB], SARC-F), and comorbidity status would represent a major advance in clinical practice. Such a tool would enable clinicians to identify patients who can safely engage in high-intensity training, those who require modified moderate-intensity programs, and those who should begin with low-intensity, balance-focused interventions. Until such a tool is developed and validated, clinicians must rely on clinical judgment, which is inherently variable and may lead to either under-treatment (due to excessive caution) or over-treatment (due to insufficient awareness of risk).

## 4 Patient stratification and individualized prescription dilemmas

### 4.1 The absence of a validated stratification framework

Effective exercise prescription for osteoporosis demands individualized stratification based on fracture risk, functional capacity, comorbidity burden, prior exercise history, and personal preferences. However, the current clinical landscape lacks a validated, evidence-based, and operationally feasible framework for such stratification. The “Too Fit to Fracture” consensus proposed a three-tier classification (Tier A: no fracture; Tier B: single fracture; Tier C: multiple fractures with pain or kyphosis) (41), but this schema is primarily descriptive, lacks validated operational criteria, and has not been prospectively tested for its predictive validity or clinical utility. The transition thresholds between tiers are subjective, and the framework does not provide specific exercise prescriptions for each tier, leaving clinicians to make individualized decisions without standardized guidance.

The UK consensus statement similarly differentiates recommendations by fracture history and frailty status (42), yet the criteria for distinguishing “frail” from “non-frail” individuals in the context of exercise prescription are not clearly defined. Existing guidelines offer directional recommendations, such as “moderate intensity for those with vertebral fractures”, but no algorithmic decision support that integrates the multiple factors that influence exercise safety and efficacy. This ambiguity is particularly problematic for clinicians without specialized training in exercise physiology or rehabilitation, who constitute the majority of osteoporosis providers in primary care, endocrinology, and geriatric medicine.

The lack of a validated stratification tool has several consequences. First, it leads to wide variability in clinical practice: some clinicians default to conservative, low-intensity recommendations for virtually all patients with osteoporosis, potentially under-treating those who could safely benefit from higher-intensity protocols. Others, influenced by the positive findings of the LIFTMOR trial, may recommend HiRIT to patients with significant comorbidities or fracture histories without adequate safety assessment. Second, it complicates the design of future RCTs, as investigators cannot easily define homogeneous risk strata for targeted enrollment. Third, it undermines patient confidence: when patients receive conflicting advice from different clinicians, they may become confused, anxious, or dismissive of exercise recommendations altogether. The development and validation of an osteoporosis-specific exercise risk stratification instrument is therefore a high priority for both clinical care and research.

### 4.2 The sex evidence gap

Another pronounced and under-recognized dilemma is the near-total absence of evidence in men. Male osteoporosis is often neglected in both clinical practice and research: men are less likely to be screened for osteoporosis, less likely to be treated if diagnosed, and dramatically underrepresented in clinical trials (8). A review identified just four studies on the influence of exercise on BMD in men over the age of 45, with no studies specifically targeting those with osteoporosis (29). The LIFTMOR-M study represents the first and currently only RCT focusing on HiRIT for older men with decreased bone mass (47). The study demonstrated that 8 months of twice-weekly supervised HiRIT improved lumbar spine BMD by 4.1% and trochanteric BMD by 2.8%, with no serious adverse events. These findings are promising and suggest that men may respond to mechanical loading in a manner qualitatively similar to women. However, a single study cannot form the basis of clinical guidelines, and the underrepresentation of male participants in the osteoporosis exercise literature mirrors the broader historical neglect of male osteoporosis.

The sex evidence gap has practical consequences. Men with osteoporosis who seek exercise advice from their clinicians are likely to receive recommendations extrapolated from female-dominated trials, without certainty that the dose-response relationships, safety profiles, and optimal modalities are equivalent. Biological sex differences in bone metabolism, muscle mass, hormonal milieu, and fracture patterns may modify the osteogenic response to exercise. For example, men have higher baseline muscle mass and may tolerate higher absolute loads, but they also have a higher prevalence of comorbidities such as cardiovascular disease that may limit exercise tolerance. The absence of sex-specific evidence means that these nuances cannot be incorporated into clinical decision-making. Future research must prioritize the inclusion of men in osteoporosis exercise trials, ideally through dedicated, adequately powered studies that address the unique clinical characteristics of male osteoporosis.

### 4.3 Comorbidity-adjusted prescription

Multimorbidity is the norm rather than the exception in older adults with osteoporosis. Cardiovascular disease, hypertension, diabetes mellitus with peripheral neuropathy, chronic obstructive pulmonary disease, osteoarthritis, joint arthroplasty, and kyphotic deformity are all common in this population and can substantially modify exercise tolerability, safety, and efficacy. The ACSM 2015 preparticipation screening algorithm, whose explicit purpose is to identify individuals at elevated risk for exercise-related sudden cardiac death and acute myocardial infarction, relies on three factors: current exercise participation, presence of signs/symptoms or known cardiovascular/metabolic/renal disease, and desired exercise intensity (48). Applied to U.S. adults ≥40 years, the algorithm would refer 2.6% to medical clearance before vigorous exercise and 54.2% before any exercise (46). However, because the algorithm’s criteria are not bone-specific, they may be overly conservative for some osteoporosis patients (e.g., those with well-controlled comorbidities) yet insufficiently specific for others at elevated fracture risk, a limitation that warrants consideration when prescribing exercise for osteoporotic populations. For example, a patient with well-controlled hypertension, no target organ damage, and no cardiovascular symptoms may be able to safely engage in high-intensity resistance training with appropriate supervision and progressive loading (ACSM frequency, intensity, time, and type (FITT) recommendations: resistance training at 60%–70% 1RM, progressing to 80% 1RM, with older adults and novices beginning at 40%–50% 1RM), a principle consistent with the American Heart Association (AHA)’s 2023 scientific statement that resistance training is safe and effective for adults with and without cardiovascular disease (49), yet the ACSM algorithm may classify this individual as requiring physician clearance, potentially creating unnecessary barriers to initiation.

Conversely, the absence of osteoporosis-specific safety data means that clinicians frequently default to conservative, lower-intensity recommendations that may be insufficient to stimulate bone adaptation. A patient with diabetes and peripheral neuropathy may be advised against high-impact exercise due to fall risk and foot injury concerns, which is reasonable, but the alternative recommendation (e.g., low-intensity walking or gentle stretching) may fall below the osteogenic threshold, rendering the exercise prescription clinically ineffective. This creates a therapeutic dilemma: the conditions that make high-intensity exercise unsafe are often the same conditions that make low-intensity exercise insufficient. The challenge is to identify the “safe and effective zone” for each patient, a task that requires individualized assessment, multidisciplinary collaboration, and often creative problem-solving (e.g., seated resistance training for patients with lower extremity limitations, water-based resistance training for patients with severe balance impairment). The evidence base for these adapted approaches is minimal, and clinical guidelines provide little guidance on how to modify exercise prescriptions for complex multimorbid patients.

### 4.4 Osteosarcopenia: an emerging dual-target challenge

Osteosarcopenia, defined as the concomitant presence of osteopenia or osteoporosis and sarcopenia, has emerged as a clinically consequential geriatric syndrome that compounds the morbidity and mortality risks associated with either condition in isolation (50). From an exercise-prescription perspective, osteosarcopenia presents a distinctive therapeutic dilemma: these patients require an adequate mechanical stimulus to simultaneously preserve bone and muscle, yet the very deficits that define the condition (reduced muscle strength, impaired physical function, and limited functional reserve) may increase susceptibility to falls, exercise-related injuries, and adverse events. Despite its clinical importance, the evidence base supporting exercise interventions specifically targeting osteosarcopenia remains remarkably sparse. A recent systematic review identified only four RCTs examining the effectiveness of physical exercise on osteosarcopenia in older adults, underscoring the paucity of high-quality intervention evidence and the immaturity of individualized prescription frameworks for this population (51).

Among the available trials, the Franconian Osteopenia and Sarcopenia Trial (FrOST) provided the most robust evidence to date. In community-dwelling older men with osteosarcopenia, 12 months of low-volume, high-intensity resistance training combined with whey protein, vitamin D, and calcium supplementation maintained lumbar spine BMD (with significant between-group differences due to a decline in the control group) and significantly improved skeletal muscle mass index and maximal hip/leg-extensor strength (52). However, changes in total hip BMD did not reach statistical significance between groups. The Korean ERTO-K trial extended this evidence to postmenopausal women, demonstrating that 6 months of progressive resistance exercise combined with nutritional support improved skeletal muscle index and handgrip strength; however, between-group differences in BMD did not reach statistical significance, a finding that may reflect the relatively short intervention duration and modest sample size (53). These trials collectively suggest that resistance training can concurrently benefit bone and muscle in osteosarcopenic individuals, but they also highlight critical unresolved questions. The optimal exercise modality, intensity progression, and minimum effective dose for simultaneous osteogenic and myogenic adaptation remain undefined. Furthermore, follow-up detraining analyses from the FrOST cohort revealed that approximately one-third of the intervention-induced gains in muscle quality were lost after 6 months of exercise cessation (54), underscoring the fragility of benefits in this population and the necessity of sustained, long-term maintenance strategies. Until larger, multi-site RCTs with fracture-powered endpoints and standardized exercise-reporting frameworks are available, exercise prescription for osteosarcopenia must rely on cautious extrapolation from osteoporosis and sarcopenia trials, with particular attention to individualized risk stratification and progressive familiarization.

## 5 The implementation chasm: from evidence to real-world practice

### 5.1 Adherence, retention, fidelity, and maintenance

Even if the efficacy of exercise for osteoporosis were unequivocally established through fracture-powered trials, its clinical utility would depend critically on sustained behavior change, a domain where the evidence is sobering and the reality is discouraging. Implementation science distinguishes four related but conceptually distinct constructs that are frequently conflated in the exercise literature. Adherence (or compliance) refers to the extent to which participants complete prescribed exercise sessions and achieve target doses. Retention refers to remaining enrolled in the study or program. Fidelity refers to whether the intervention is delivered and performed as intended, including correct intensity, technique, and progression. Maintenance refers to continued exercise behavior after the structured intervention ends. These constructs are not interchangeable: a dropout reflects a retention failure, not necessarily poor adherence; conversely, high attendance does not guarantee adequate intensity or progression if fidelity is compromised. A systematic review of 30 RCTs examining exercise in individuals with osteopenia or osteoporosis found that participants adhered to 83% (range 59%–100%) of prescribed exercise sessions within the structured trial environment, yet only 76% of included studies clearly reported adherence rates, and the methods for promoting and measuring adherence were often inadequately described, preventing meta-analytic pooling (55). Notably, qualitative studies have identified “fear of injury” or “fear of falling” as the most significant psychological barrier in osteoporosis populations (56), yet this factor was mentioned in only two of the 30 RCTs included in a recent systematic review, reflecting a broader methodological gap wherein RCTs primarily reported physiological outcomes and adherence metrics without validated psychological tools to measure fear of movement (55).

Adherence to prescribed exercise parameters is not merely a behavioral preference but a biological determinant of osteogenic efficacy. A systematic review of 32 RCTs compared exercise interventions with high versus low adherence to ACSM recommendations in individuals with osteoporosis (57). High-adherence programs produced significantly larger improvements in BMD than low- or uncertain-adherence programs at both the lumbar spine (SMD: 0.31 *vs.* 0.04) and femoral neck (SMD: 0.45 *vs.* 0.28). These findings underscore that the therapeutic value of exercise depends not only on whether a patient exercises, but on whether the prescribed dose, including frequency, intensity, duration, and type, is actually delivered and performed as intended.

The mode of exercise delivery matters profoundly for both adherence and efficacy. Meta-analytic evidence suggests that supervised, group-based exercise programs are associated with approximately twice the fracture-prevention effect observed in predominantly unsupervised home-based programs (28). This difference likely reflects the combined influence of multiple correlated factors: supervision ensures correct intensity, technique, and progression; group settings provide social support, accountability, and motivation; and supervised trials typically enroll more motivated participants with fewer barriers to attendance. However, this estimate derives from subgroup comparisons across trials rather than from a formal randomized comparison of supervised versus unsupervised delivery. Consequently, supervision should be regarded as an important potential effect modifier rather than as a definitively established independent causal determinant of fracture reduction. Moreover, supervised programs demand specialized personnel (exercise physiologists, physiotherapists, or certified trainers), accessible facilities, and transportation, all resources that are scarce in many healthcare systems, particularly in rural, low-income, and lower-resource settings. Patients who are motivated to exercise often lack guidance on how to do so safely and effectively, while those who are referred to generic community exercise programs may receive instruction that is insufficiently specific to their bone health needs.

A further barrier to real-world implementation is that published exercise protocols are frequently reported with insufficient detail to permit faithful replication. A systematic review of 39 HiRIT studies found that while core exercise components were generally well described, important elements, including loading progression algorithms, individualization criteria, home-based exercise components, and motivational or behavioral support strategies, were recurrently omitted (39). Such reporting deficiencies mean that even clinicians who wish to replicate efficacious trial protocols may lack the necessary information to do so safely and effectively. Without explicit guidance on how to progress load, when to modify exercises for individual patients, or how to sustain motivation after supervised contact ends, real-world fidelity is likely to fall substantially below trial standards, contributing to the implementation chasm depicted in **Figure 3**. **Figure 3** depicts these implementation chasms across six core domains, including supervised delivery, session adherence, expert supervision, facility accessibility, cost coverage, and long-term maintenance, and the divergence between the idealized conditions of RCTs and the far more constrained reality of clinical practice. Each domain is rated qualitatively, revealing that RCTs typically operate under optimal or near-optimal conditions for supervised delivery, session adherence, expert supervision, facility accessibility, and cost coverage, whereas real-world practice falls to low or minimal levels across the majority of these same domains. The gap is particularly pronounced for supervised delivery and expert supervision, domains where the absence of infrastructure, trained personnel, and reimbursement mechanisms creates a structural barrier that prevents evidence-based exercise from reaching the patients who stand to benefit most.

**Figure 3:**
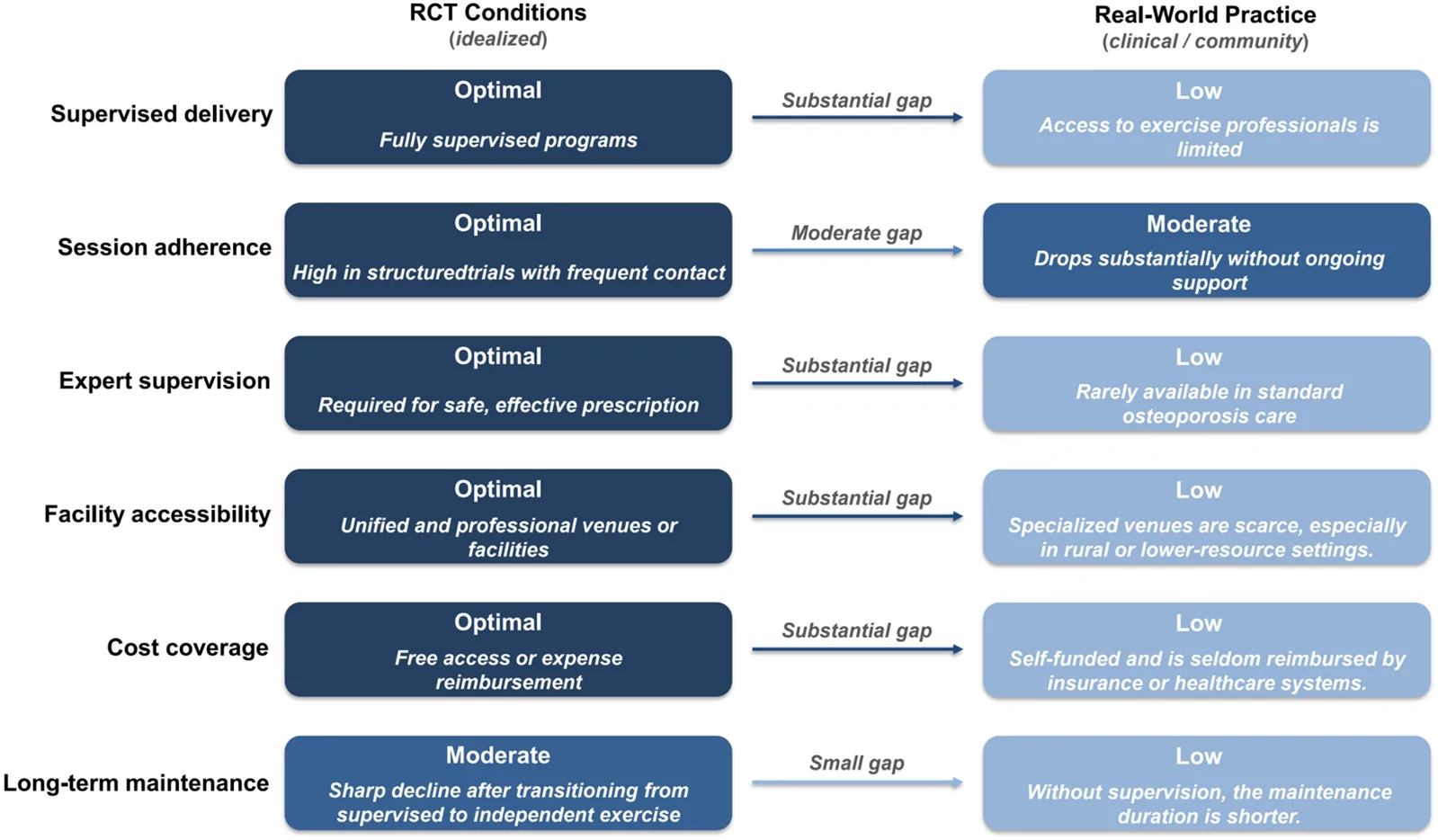
Conceptual representation of the implementation chasm between idealized randomized controlled trial (RCT) conditions and real-world exercise therapy for osteoporosis. The left bar represents the typical condition under which RCTs are conducted, and the right bar represents the corresponding condition in real-world clinical or community settings. This figure presents a qualitative conceptual framework, not quantified estimates. The ratings for each of the six implementation domains (supervised delivery, session adherence, expert supervision, facility accessibility, cost coverage, and long-term maintenance) were assigned by the authors based on the consensus reported in systematic reviews of exercise adherence (55, 58) and international consensus statements (42).

The temporal dynamics of exercise behavior are also problematic. Adherence to structured programs is typically highest during the initial weeks, declines gradually over the first 3–6 months, and stabilizes at a lower plateau for those who remain enrolled. Long-term retention data from real-world adapted physical activity programs show this attrition starkly: follow-up attendance fell from 81% at 2 months to 27% at 6 months and 18% at 12 months after cardiac rehabilitation (59). Maintenance, the continuation of exercise after the supervised program ends, represents a distinct and even more challenging phase: without ongoing support, monitoring, and reinforcement, many patients revert to pre-intervention activity levels within months. The transition from supervised to independent exercise is therefore a critical vulnerability point. The reasons for this decline are multifactorial and include loss of novelty, fading motivation, competing time demands, physical discomfort, perceived lack of benefit, and the absence of a structured routine.

Implementation science research suggests that sustained behavior change requires not only initial education and skill acquisition but also ongoing self-monitoring, feedback, goal-setting, and problem-solving support. A meta-analysis of 45 studies demonstrated that multi-component goal setting interventions, which incorporate feedback, strategy planning, and rewards alongside goal setting, produce a medium, positive effect on physical activity behavior (60). However, the behavior change techniques identified as critical for sustained change, including self-monitoring, goal setting, feedback, and problem-solving, are seldom systematically delivered in standard clinical care. A systematic review found that healthcare professionals across disciplines consistently report perceived lack of time, lack of prioritization for health behavior change, and insufficient skills as barriers to delivering behavior change interventions (61). Sustained change also requires enhancing patients’ internal motivation and creating a supportive environment, elements rarely embedded in routine care (62). This implementation gap underscores why the supervised-to-independent transition is a critical vulnerability point for patients with osteoporosis.

### 5.2 Detraining and the fragility of bone gains

The consequences of failed maintenance extend beyond mere non-adherence to encompass the active loss of previously acquired benefits. When patients discontinue or substantially reduce osteogenic loading, bone tissue does not simply plateau. Bone is a dynamic tissue that responds continuously to mechanical cues, and the cessation or substantial reduction of osteogenic loading triggers a rapid shift toward net bone resorption. The ACTLIFE study, a German randomized controlled trial, demonstrated that 3 months of detraining following 13 months of supervised HiRIT attenuated the lumbar spine BMD gains: the previously significant between-group difference was no longer statistically significant (p = 0.065) (63). A longer-term follow-up by Englund and colleagues, conducted 5 years after the cessation of a 1-year exercise program, found that all bone gains had completely disappeared, with hip BMD in the former exercise group falling below that of the control group, suggesting that the transient stimulation of bone formation may have been followed by a compensatory period of accelerated resorption (64).

These findings have profound implications for clinical practice and public health messaging. They underscore that exercise for osteoporosis is not a finite “course” or “treatment program” but a lifelong behavior that must be sustained to maintain benefits. Yet clinical practice continues to frame exercise as a rehabilitative intervention with a defined start and end date, and patients often perceive exercise prescriptions as temporary commitments rather than permanent lifestyle modifications. There is little infrastructure to support sustained adherence: most supervised exercise programs last 6–12 months, after which participants are transitioned to independent or unsupervised maintenance, with minimal follow-up and no systematic monitoring of bone status. The optimal maintenance dose of exercise, whether through reduced frequency (e.g., once weekly rather than twice weekly), reduced intensity (e.g., 60% 1RM rather than 80% 1RM), or alternative modalities (e.g., replacing impact training with brisk walking), has not been empirically determined. This evidence gap leaves clinicians and patients without guidance on how to sustain bone health after the initial intensive phase, and it contributes to the high rates of bone loss and fracture recurrence observed in the years following exercise program cessation.

### 5.3 The clinical environment gap

The implementation gap is not merely a patient-level adherence problem but a systemic one that reflects the organization, financing, and culture of healthcare delivery. Osteoporosis care is typically fragmented across multiple disciplines: endocrinology manages pharmacological therapy; orthopedics handles fractures and surgical interventions; geriatrics addresses multimorbidity and functional decline; rehabilitation provides post-fracture physical therapy; and primary care serves as the entry point but often lacks the expertise or time to manage complex exercise prescriptions. No single discipline assumes comprehensive responsibility for exercise prescription, monitoring, and progression, and communication between disciplines is often poor. The concept of a Fracture Liaison Service (FLS) has been promoted as a model to bridge this gap, but FLS implementation remains inconsistent across countries and healthcare systems, and the exercise component of the FLS is often underdeveloped, with services focusing primarily on pharmacological initiation and bone density monitoring rather than on long-term exercise behavior change (65).

Moreover, the reimbursement landscape in most countries favors pharmacological interventions over non-pharmacological therapies. Exercise therapy, despite its multi-system benefits (improved cardiovascular health, metabolic regulation, mental health, functional independence, and falls prevention), rarely receives comparable insurance coverage or institutional support. In many healthcare systems, exercise programs are classified as “wellness” or “preventive services” rather than as medical treatments, and they are not reimbursed at rates that would sustain the infrastructure required for high-quality, supervised, ongoing delivery (66, 67). This structural imbalance reinforces the perception among both clinicians and patients that exercise is an adjunctive recommendation rather than a core therapeutic modality. Until healthcare financing models evolve to reimburse exercise therapy as a legitimate and essential component of osteoporosis management, the implementation gap will persist regardless of how compelling the efficacy evidence becomes.

### 5.4 Communication dilemmas

Clinicians face a profound communication dilemma when counseling patients about exercise for osteoporosis. On one hand, overly general advice (“do more exercise,” “walk more often”) is ineffective because it provides no actionable specificity: patients do not know what type of exercise, what intensity, what frequency, or what duration is appropriate for their bone health needs (68). Such vague recommendations may lead to counterproductive behaviors, such as excessive walking (which has minimal osteogenic effect and may exacerbate joint pain in patients with osteoarthritis) or avoidance of all loading activities (which accelerates bone loss). On the other hand, overly detailed HiRIT prescriptions, specifying exact percentages of 1RM, sets, repetitions, and progression algorithms, may intimidate patients, particularly those with fracture anxiety, low self-efficacy, limited health literacy, or cultural unfamiliarity with gym-based resistance training. The UK consensus emphasizes a “how to” rather than “don’t do” messaging approach (42), but delivering this nuance requires time, expertise, and rapport that are often unavailable in brief clinical encounters lasting 10–15 minutes.

Qualitative research indicates that patients with osteoporosis frequently receive conflicting, absent, or discouraging exercise advice from health professionals (68–70). In a study by Ziebart and colleagues, participants reported that clinicians often advised them to “be careful” without providing constructive guidance on what they could safely do, which paradoxically increased anxiety and activity avoidance (56). Some patients were explicitly told to avoid lifting, bending, or vigorous activity, a message that, while well-intentioned, can lead to deconditioning, muscle atrophy, and accelerated bone loss. The ideal communication strategy balances reassurance with specificity, empowers patients with actionable knowledge, and acknowledges uncertainty where it exists. However, training clinicians in exercise counseling is rarely part of medical or nursing education, and most osteoporosis providers lack the practical skills to demonstrate exercises, assess technique, or design progressive programs. This educational gap is a fundamental barrier to effective clinical translation.

## 6 Nutritional cofactors as unmet prerequisites

### 6.1 The nutrition–exercise interaction

The skeletal response to mechanical stress is often overlooked but is critically dependent on proper nutritional support. Bone remodeling is an energetically expensive and materially demanding process that requires sufficient protein for matrix synthesis (type I collagen constitutes approximately 90% of the organic bone matrix), calcium for mineralization, and vitamin D for intestinal calcium absorption and neuromuscular function (71, 72). The IOF and the European Society for Clinical and Economical Aspects of Osteoporosis, Osteoarthritis, and Musculoskeletal Diseases (ESCEO) recommend a protein intake of at least 1.0–1.2 g/kg/day and a calcium intake of at least 1,000–1,200 mg/day for older adults to maintain bone health (73). These recommendations should be individualized according to renal function, chronic kidney disease stage, and overall nutritional status; blanket increases in protein intake may be inappropriate or even harmful for patients with impaired renal clearance. However, population-level data reveal alarming deficiencies that are widespread and often unrecognized.

In China, a country with the world’s largest aging population, the median dietary calcium intake among adults is approximately 330 mg/day, less than half of the Estimated Average Requirement (EAR: 650 mg/day for ages 18–49 and 800 mg/day for ages 50–64), with dairy products contributing only 6.7% of total intake (74). This deficiency is attributable to cultural dietary patterns (low dairy consumption, limited fortified foods), lactose intolerance prevalence, and socioeconomic factors affecting access to calcium-rich foods. Dietary protein intake among Chinese older adults declined to a median of just 57.8 g/day in 2018, leaving 44% of the population below the estimated average requirement and fewer than half (47.3%) exceeding the recommended nutrient intake (RNI; 1.0 g/kg/day), with the deficit most severe in those aged ≥70 years, those with low levels of education, and those in rural areas (75). Similar patterns are observed in other East Asian populations, where traditional diets are often low in both dairy and high-quality protein sources. These nutritional deficits are not confined to Asia: in the United States and Europe, substantial proportions of older adults consume inadequate calcium and protein, particularly those with limited income, social isolation, or impaired appetite.

When exercise is prescribed without addressing underlying nutritional deficits, its osteogenic potential may be systematically attenuated. A study found that protein supplementation combined with resistance training yields only marginal additional BMD benefits compared to exercise alone (76), but this finding reflects the methodological limitation that most trials enroll participants with protein intakes that are already near or above the threshold for adequacy. In reality, however, the majority of older adults in many populations begin exercise from a state of insufficient nutrition. Therefore, no reliable conclusion can yet be drawn as to whether combining protein and exercise interventions confers greater skeletal benefits than either component alone. Nevertheless, several studies have indicated that protein intake exceeding the Recommended Dietary Allowance (RDA) may increase BMD and bone mineral content (77, 78). In this context, nutritional intervention may be as critical as the exercise stimulus itself, yet nutritional assessment is rarely integrated into exercise prescription pathways. Clinicians should verify calcium, protein, and vitamin D status and assess renal function before or concurrently with exercise initiation, and should refer to dietitians when deficits are identified or when protein recommendations need to be modified for patients with chronic kidney disease.

### 6.2 Vitamin D: sufficiency versus supraphysiological dosing

Vitamin D occupies a particularly complex position in the exercise-osteoporosis relationship. While vitamin D deficiency impairs muscle function, bone mineralization, and calcium absorption (79), evidence indicates that vitamin D and exercise exert largely independent effects on falls and BMD, and that their interaction is more nuanced than previously assumed. The DO-HEALTH trial, a large, multicenter RCT conducted in five European countries, found that vitamin D supplementation at 2,000 IU/day improved total hip BMD slightly (0.4%), but the simple home exercise program (SHEP) did not, and critically, there was no interaction between the two interventions: the benefits of vitamin D and exercise were additive, not synergistic (80). It is important to recognize, however, that the exercise intervention in DO-HEALTH was a low-intensity, minimally progressive home-based program rather than a structured, supervised, progressive HiRIT intervention. The original investigators themselves highlighted the low intensity and limited progression of SHEP as potential explanations for its lack of effect on aBMD. Consequently, the absence of a vitamin D–exercise interaction in DO-HEALTH should not be generalized to all exercise modalities.

A 2024 network meta-analysis of vitamin D supplementation for fall prevention further clarified the dose-response relationship: vitamin D fall-prevention benefits were confined to individuals with serum 25-hydroxyvitamin D [25(OH)D] levels ≤ 50 nmol/L, and doses exceeding 1,000 IU/day may paradoxically increase fall risk in older adults, possibly through adverse effects on muscle function or gait stability at supraphysiological concentrations (81). These observations should be interpreted cautiously: they derive from a single network meta-analysis of heterogeneous trials with varying dosing regimens (daily, weekly, monthly), baseline vitamin D status distributions, population characteristics, and outcome definitions. The apparent risk signal at higher doses may not apply uniformly to all supplementation schedules or to individuals with substantially different baseline status. Nevertheless, these findings suggest that the blanket co-prescription of high-dose vitamin D (often 2,000–4,000 IU/day) with exercise is not supported by current evidence; a test-and-treat approach based on baseline serum 25(OH)D levels is more rational and potentially safer. Individuals with levels < 30 nmol/L should be repleted, whereas those with levels ≥ 50 nmol/L are unlikely to derive additional bone or fall-prevention benefit from further supplementation in the contexts studied, though this threshold may vary with dosing regimen and target outcome. However, this individualized approach is rarely operationalized in routine clinical practice, where many clinicians continue to prescribe fixed high-dose vitamin D regimens without biochemical assessment.

## 7 Traditional mind-body exercises within modern evidence frameworks

### 7.1 Evidentiary positioning

Tai Chi, Baduanjin (Eight Pieces of Brocade), Wuqinxi (Five Animals Play), and Yijinjing (Muscle-Tendon Change Classic) are widely practiced in East Asian communities and are increasingly recommended as osteoporosis-friendly exercise options by clinicians and traditional medicine practitioners (82). Their appeal is multifaceted: they require no equipment, minimal space, can be performed indoors or outdoors, are culturally familiar and socially acceptable, and combine gentle physical movement with elements of mindfulness, breath control, and stress reduction. These characteristics may be particularly important for older adults who are reluctant to engage in gym-based resistance training, who lack access to facilities, or who have vertebral fractures limiting high-load exercise.

Recent meta-analyses have demonstrated statistically significant BMD improvements with Tai Chi at the lumbar spine, femoral neck, and greater trochanter (83). A network meta-analysis of 31 RCTs that included only traditional Chinese fitness exercises reported that Baduanjin combined with drug interventions received the highest surface under the cumulative ranking curve (SUCRA) values for lumbar spine BMD (83.6%) and femoral neck BMD (90.2%) (84). Importantly, because this network did not include resistance training, high-impact exercise, or combined protocols, these rankings do not permit cross-modality comparison with the osteogenically superior interventions presented in **Figure 1**. However, SUCRA rankings are probabilistic estimates that reflect relative performance within a specific network and are sensitive to network consistency, evidence sparsity, and baseline risk. They should not be interpreted as definitive evidence that one modality is clinically superior to another, particularly when the underlying evidence is characterized by small samples, risk of bias, inconsistency, and methodological heterogeneity.

The BMD benefits of Tai Chi are primarily observed when the comparator is no exercise or usual care, and the effect sizes are modest in comparison with those achieved by progressive resistance training or combined resistance and impact training (27). When Tai Chi is compared head-to-head with progressive resistance training, the differences are not statistically significant. However, these direct comparisons are typically underpowered and were not designed or powered to demonstrate equivalence or non-inferiority. Consequently, the absence of a statistically significant difference should not be interpreted as evidence that Tai Chi and progressive resistance training exert comparable osteogenic effects. It is therefore premature to conclude that the observed BMD benefits are attributable to physical activity per se rather than to any Tai Chi-specific mechanism (85). Moreover, the methodological quality of traditional exercise RCTs is generally low: randomization procedures are frequently unclear, outcome assessors are rarely blinded, intention-to-treat analysis is inconsistently applied, and trial registration is not universally reported. An umbrella review by Li and colleagues graded the overall certainty of evidence for Tai Chi on BMD as “low” according to the Grading of Recommendations Assessment, Development and Evaluation (GRADE) framework (86), reflecting the combined limitations of risk of bias, inconsistency, and indirectness.

The apparent contradiction between the positive findings of individual studies and the low certainty of the overall evidence base highlights a broader methodological challenge in the traditional exercise literature. Many studies are conducted in China and published in Chinese-language journals, which may introduce publication bias and limit accessibility to international reviewers. The heterogeneity of Tai Chi styles (e.g., Yang, Chen, Sun, Wu), training durations, frequencies, and instructor qualifications further complicates synthesis. Some forms of Tai Chi emphasize slow, continuous movement with minimal loading, while others incorporate more dynamic weight shifting and lower-extremity strengthening. Without standardized reporting of these parameters, clinicians cannot determine which style, duration, and intensity are most osteogenic. The field would benefit from a core outcome set for traditional exercise trials, analogous to those developed for pharmacological trials, to improve comparability and evidence quality.

### 7.2 Clinical utility and cultural context

Despite the evidentiary limitations discussed above, traditional exercises occupy an important and pragmatic niche in osteoporosis care that should not be dismissed. For older adults who are reluctant to engage in gym-based resistance training, who lack access to supervised facilities, or who have vertebral fractures limiting high-load exercise, Tai Chi and Baduanjin offer culturally familiar, low-risk alternatives with established benefits for balance, falls prevention, and psychological well-being. The Cochrane review by Sherrington and colleagues, which included 108 RCTs, reported that Tai Chi reduces fall rates by 23% (rate ratio 0.77, 95% CI: 0.71–0.83) with moderate-certainty evidence (87), a finding that exceeds its BMD evidence in both robustness and clinical relevance. For fall-prone older individuals, the fracture-prevention value of Tai Chi via balance improvement may well outweigh its modest BMD effects, particularly given that falls are the proximal cause of approximately 90% of hip fractures and many vertebral fractures.

The dilemma lies in how these modalities are positioned within clinical guidelines and communicated to patients. The 2025 Chinese guideline for non-pharmacological intervention recommends Tai Chi, Baduanjin, Wuqinxi, and Yijinjing as evidence-based options for improving BMD and balance, with recommendation grades ranging from 2C to 1A (88). However, the guideline acknowledges that these recommendations are based primarily on BMD surrogate endpoints, not fracture outcomes, and that the evidence for resistance training remains stronger and more consistent. Clinicians must therefore balance cultural acceptability and adherence advantages against the empirically superior osteogenic potential of HiRIT. In practice, this often means presenting traditional exercises as complementary rather than as substitutes: a patient might engage in Tai Chi for balance and fall prevention while simultaneously performing supervised resistance training for bone density. This dual-modality approach has intuitive appeal but has not been formally tested in RCTs, and the feasibility of sustaining two concurrent exercise programs in real-world settings is uncertain.

Cultural context also matters. In East Asian populations, traditional exercises are deeply embedded in community life and are often practiced in parks, community centers, and senior groups. This social infrastructure may facilitate adherence in ways that gym-based programs cannot replicate. Conversely, in Western populations, where Tai Chi is less familiar and may be perceived as “alternative” rather than mainstream, recruitment and adherence may be more challenging. The cultural specificity of exercise preferences underscores the need for patient-centered, shared decision-making in exercise prescription, rather than the imposition of a single “best” modality. What is optimal for one patient may be suboptimal for another, and the evidence base must be interpreted through the perspective of individual circumstances, values, and resources.

## 8 Future directions and conclusions

The landscape of exercise therapy for osteoporosis is characterized by a fundamental paradox: the biological rationale is robust, the consensus recommendations are broadly consistent, and yet the clinical execution is fraught with uncertainty, structural barriers, and persistent gaps between what is known and what is done. This review has identified and examined seven principal challenges that collectively define this paradox: the surrogate endpoint dependency of the evidence base, the heterogeneity of outcomes across modalities, the intensity paradox, the absence of validated stratification tools, the implementation chasm, the nutritional inadequacies that frequently accompany exercise prescription, and the methodologically heterogeneous evidence for traditional exercises. These challenges are not arguments against the use of exercise in osteoporosis; they are arguments for more honest, nuanced, and resource-intensive engagement with it.

Several strategic priorities emerge from this analysis. The field must move beyond BMD as the sole arbiter of efficacy and pursue fracture-powered trials. Although logistically challenging, pragmatic trials leveraging existing cohorts, Fracture Liaison Service infrastructure, or large health system databases could provide real-world fracture data with exercise interventions. Hybrid effectiveness-implementation trials that simultaneously evaluate clinical outcomes and delivery strategies may be particularly valuable, provided they incorporate standardized exercise protocols reported using validated frameworks such as the Consensus on Exercise Reporting Template (CERT), validated adherence monitoring, and long-term follow-up to assess both efficacy and sustainability.

Validated pre-exercise stratification tools specific to osteoporosis must also be developed and prospectively tested. The current reliance on generic cardiovascular screening algorithms and expert-opinion fracture-risk categories is inadequate for individualized exercise prescription. An instrument integrating bone density, fracture history, vertebral morphology (Genant grading), functional capacity (TUG, SPPB), sarcopenia screening (SARC-F), and comorbidity status would enable safer, more precise intensity titration. Such a tool should be validated across diverse populations, including men, different ethnic groups, and varying healthcare settings, to ensure generalizability.

Implementation science must address the adherence-to-supervision gap. Digital health technologies, remote exercise monitoring via wearable sensors, app-based coaching with automated feedback, virtual reality balance training, and telehealth consultations with exercise professionals may offer scalable alternatives to in-person supervision, though their efficacy, safety, and cost-effectiveness in osteoporosis populations require formal testing (89). Simultaneously, healthcare financing models must evolve to reimburse exercise therapy as a core component of osteoporosis management rather than as an ancillary lifestyle suggestion. Bundled payment models that include exercise therapy within osteoporosis care pathways may incentivize healthcare systems to invest in the necessary infrastructure.

Nutritional assessment and optimization should be integrated into exercise prescription pathways at exercise initiation, rather than treated as an afterthought. In populations with endemic calcium and protein insufficiency, nutritional intervention may be as critical as the exercise stimulus itself. Integrated diet-exercise pathways, potentially co-managed by dietitians and exercise professionals within multidisciplinary osteoporosis clinics, should be developed and evaluated. The role of vitamin D should be individualized based on serum 25(OH)D levels, with routine supplementation reserved for deficient individuals rather than applied indiscriminately.

The current near-total absence of male-specific exercise trial data represents a scientific and clinical failing that demands redress. Dedicated trials in men with osteoporosis or osteopenia should assess the efficacy, safety, and optimal modalities of exercise, taking into account the biological and clinical differences between male and female osteoporosis. The LIFTMOR-M trial provides a valuable template, but larger, longer, and more diverse studies are needed.

The majority of exercise RCTs have enrolled peri- and postmenopausal women with osteopenia or low bone mass, generally healthy community-dwelling older adults, or mixed populations with broadly defined ‘low bone mass’ rather than individuals with established osteoporosis, severe fragility fractures, advanced frailty, or major comorbidity. Trials such as LIFTMOR explicitly excluded participants with uncontrolled cardiovascular disease, severe mobility limitation, or recent fractures (15), and DO-HEALTH recruited only individuals without major health events in the preceding 5 years (80). Consequently, the efficacy and safety profiles that underpin current exercise recommendations cannot be assumed to generalize automatically to the highest-risk segments of the osteoporosis population—those with T-scores ≤ −3.0, multiple vertebral fractures, substantial kyphotic deformity, or complex multimorbidity. For these individuals, exercise prescription remains largely empirical, informed by expert consensus rather than by direct evidence. Dedicated trials in severe osteoporosis and high-fracture-risk populations are urgently needed to determine optimal modality, intensity, progression, and safety parameters.

The evidentiary base for traditional mind-body exercises must be strengthened through higher-quality RCTs. Future studies should employ robust randomization, blinded outcome assessment, intention-to-treat analysis, and standardized reporting of exercise parameters (style, duration, frequency, instructor qualifications). Head-to-head comparisons with progressive resistance training, rather than comparisons with no exercise, are essential to determine whether traditional exercises offer any unique benefits beyond physical activity per se. Core outcome sets specific to traditional exercise trials would improve comparability and synthesis.

In conclusion, exercise therapy remains one of the most promising yet most fragile interventions in osteoporosis care. Its efficacy is contingent not only on the correct mechanical stimulus but on a complex interplay of patient stratification, nutritional sufficiency, behavioral adherence, and systemic support. The challenges outlined in this review are not reasons to abandon exercise; they are reasons to pursue it more intelligently, more honestly, and with greater investment in the research, education, and infrastructure necessary to realize its full potential. Clinicians should continue to recommend exercise while acknowledging the limits of current evidence, individualizing prescriptions to the extent possible, and advocating for the multidisciplinary resources and policy changes necessary to close the gap between what exercise can do in theory and what it currently achieves in practice. As the global burden of osteoporosis continues to grow, the imperative to optimize non-pharmacological interventions becomes ever more urgent. Exercise is not a universal cure, but it is a powerful tool that, when properly applied, can meaningfully improve the lives of millions of older adults at risk of fragility fractures. The question is no longer whether appropriately prescribed exercise can improve relevant determinants of bone and functional health, but how to optimize its type, dose, safety, sustainability, and implementation for different individuals across the osteoporosis risk spectrum.
