# Supplementary material for: Challenges and dilemmas of exercise therapy in the management of osteoporosis: a narrative review
Source: Front Endocrinol (Lausanne). 2026 Sep 29;17:1931476. doi: 10.3389/fendo.2026.1931476 (PMC13658988; doi:10.3389/fendo.2026.1931476)
Supplement: Supplementary file 1 [file Table1.docx]

Table S1. Source characteristics and effect estimates for exercise modalities presented in Figure 1.

| **Exercise modality** | **Samples (n)** | **Femoral neck** | | **Lumbar vertebra** | |
| --- | --- | --- | --- | --- | --- |
|  |  | **MD (95% CI)** | **P-score** | **MD (95% CI)** | **P-score** |
| **AE + RT** | 135 | 140.00 (40.89; 239.11) | 0.99 | 32.35 (8.08; 56.62) | 0.87 |
| **WBV** | 127 | 26.07 (2.97; 49.16) | 0.8 | 22.33 (6.67; 37.99) | 0.74 |
| **RT** | 365 | 16.98 (8.98; 24.99) | 0.72 | 17.16 (−5.61; 39.94) | 0.6 |
| **Tai Chi** | 110 | 9.14 (−15.88; 34.16) | 0.47 | 16.38 (5.03; 27.73) | 0.6 |
| **AE** | 253 | 7.53 (−4.12; 19.19) | 0.44 | 10.61 (−0.01; 21.23) | 0.44 |
| **Mixed Exercise** | 708 | 5.24 (−2.55; 13.02) | 0.38 | 0.06 (−99.68; 99.79) | 0.4 |
| **Impact Exercise** | 100 | 3.01 (−17.79; 23.80) | 0.32 | 4.28 (−33.85; 42.41) | 0.35 |
| **Walking** | 106 | −0.19 (−16.44; 16.06) | 0.22 | 6.11 (−9.97; 22.19) | 0.33 |
| **Control** | 1456 | 0 | 0.17 | 0 | 0.16 |

MD: Mean Difference, CI: Credible Interval, AE + RT: Aerobic Mixed Resistance Exercise, WBV: Whole Body Vibration, RT: Resistance Training, AE: Aerobic Exercise, MIX: Mixed Exercise, IE: Impact Exercise.
